# In Silico Analysis of Publicly Available Transcriptomic Data for the Identification of Triple-Negative Breast Cancer-Specific Biomarkers

**DOI:** 10.3390/life13020422

**Published:** 2023-02-02

**Authors:** Rachid Kaddoura, Fatma Alqutami, Mohamed Asbaita, Mahmood Hachim

**Affiliations:** College of Medicine, Mohammed Bin Rashid University of Medicine and Health Sciences, Dubai P.O. Box 505055, United Arab Emirates

**Keywords:** triple-negative breast cancer, in silico analysis, differentially expressed gene, biomarkers, GATA 3, FOXA1, tumor microenvironment

## Abstract

Background: Breast cancer is the most common type of cancer among women and is classified into multiple subtypes. Triple-negative breast cancer (TNBC) is the most aggressive subtype, with high mortality rates and limited treatment options such as chemotherapy and radiation. Due to the heterogeneity and complexity of TNBC, there is a lack of reliable biomarkers that can be used to aid in the early diagnosis and prognosis of TNBC in a non-invasive screening method. Aim: This study aims to use in silico methods to identify potential biomarkers for TNBC screening and diagnosis, as well as potential therapeutic markers. Methods: Publicly available transcriptomic data of breast cancer patients published in the NCBI’s GEO database were used in this analysis. Data were analyzed with the online tool GEO2R to identify differentially expressed genes (DEGs). Genes that were differentially expressed in more than 50% of the datasets were selected for further analysis. Metascape, Kaplan-Meier plotter, cBioPortal, and the online tool TIMER were used for functional pathway analysis to identify the biological role and functional pathways associated with these genes. Breast Cancer Gene-Expression Miner v4.7 was used to validify the obtained results in a larger cohort of datasets. Results: A total of 34 genes were identified as differentially expressed in more than half of the datasets. The DEG GATA3 had the highest degree of regulation, and it plays a role in regulating other genes. The estrogen-dependent pathway was the most enriched pathway, involving four crucial genes, including GATA3. The gene FOXA1 was consistently down-regulated in TNBC in all datasets. Conclusions: The shortlisted 34 DEGs will aid clinicians in diagnosing TNBC more accurately as well as developing targeted therapies to improve patient prognosis. In vitro and in vivo studies are further recommended to validate the results of the current study.

## 1. Introduction

Breast cancer is one of the most common types of cancer amongst women, with a very complex pathophysiology and 2.3 million newly identified cases globally in the year 2020, and a total of 7.8 million diagnoses by the end of that year [1]. Breast cancer is most commonly classified based on the molecular subtypes, which are dependent on the molecular profiles of the estrogen receptor (ER), progesterone receptor (PR), and human epidermal growth factor recpetor (HER2) [2]. Of the different molecular subtypes, the triple-negative breast cancer (TNBC) subtype is negative for all these receptors, accounts for 15–25% of the cases, and is considered to be the most aggressive subtype [3]. In the United States, TNBC has been found to yield a low five-year survival rate, of 8–16%, in comparison to the other molecular sybtypes [3].There has been a gradual increase in the incidence of breast cancer annually, with management of the disease being dependent on enhancing the outcome and survival of patients through early detection and diagnosis [4].

Current diagnostic procedures include imaging and immunohistochemistry, which aid in subtyping and classifying the disease for enhancing treatment options [5]. Recent technical developments in the transcriptomic and genomic profiling of tumors have shifted the traditional clinicopathological classification into an advanced classification based on subtyping, which demonstrated prognostic and therapeutic features [6]. Furthermore, the introduction of a minimally invasive procedures, such as liquid biopsies, can potentially increase the rate of early diagnosis as opposed to a more demanding and less appealing option—the solid biopsy. In a study that involved newly diagnosed patients, for example, the “predictive value” of plasma ddPCR using liquid biopsy for both primary EGFR mutation and KRAS mutation was 100 percent, meaning that patients who tested positive for either mutation carried said mutation in their tumor [7]. This screening accuracy, paired with the minimally invasive nature of liquid biopsies, could aid in introducing screening tests as a more common procedure, especially for situations such as of ruling out triple-negative breast cancers [7].

There is a clear distinction in the protein expression levels between the molecular subtypes of breast cancer—Luminal A, Luminal B, and HER2-enriched breast cancer—which is not present in TNBC. This lack of a precise molecular mechanism to explain TNBC limits treatment plans to the likes of chemotherapy, with an ambiguity in the levels of protein expression that are detrimental to TNBC diagnosis. Due to the disease complexity and heterogeneity, TNBC cannot be treated as a single entity, and there is no single biomarker that can be used for diagnosis, making it difficult for early recognition and prognosis [8,9]. To date, no clinical tools have been identified to easily assess whether the patient will respond to standard breast cancer treatment or have resistant de novo mutations in TNBC subtypes [9]. Therefore, there has been an increase in the drive to obtain reliable and accurate biomarkers to aid in the early detection and prognosis of TNBC, which is the motivation behind the conduction of this study.

Furthermore, in recent years, many immune cells have been found in the tumor microenvironment, each playing a different role. These different immune cells can be used as either biomarkers for tumor classification or potential therapeutic targets. For example, recruitment of tumor-associated macrophages is a potential target for tumor treatments in breast cancer [10]. Similarly, the proportion of immune cells in the tumor microenvironment can not only predict but also explain a patient’s outcome and prognosis [11].

A comprehensive understanding of the molecular changes in TNBC might identify new players that can explain the pathogenesis and serve as potential and reliable markers, which is another incentive of this study. Output omics databases and patient datasets that are publicly available, as used in this study, are an excellent source for identifying such markers.

Breast cancer patients’ expression profiles were re-analyzed after grouping them into TNBC and non-TNBC groups within their respective datasets. The aim of this study was to identify consistently differentially expressed genes (DEGs)—genes found in more than 50% of datasets—and their pathways, as well as potential patient impact. The shortlisted genes will aid clinicians in diagnosing TNBC more accurately as well as developing targeted therapies to improve patient prognosis.

## 2. Materials and Methods

### 2.1. Publicly Available Breast Cancer Transcriptomic Datasets

In order to identify consistently differentially expressed genes specific to TNBC compared to other types of breast cancer, we explored the publicly available transcriptomics data repository of the National Center for Biotechnology Information (NCBI), the Gene Expression Omnibus (GEO) (https://www.ncbi.nlm.nih.gov/geo/, accessed on 2 January 2023)—a genomic data repository—for datasets of patients with breast cancer. For consistency, we selected publicly available datasets which can be analyzed using GEO2R, a built-in platform within NCBI GEO, to carry out differential gene expression analysis on microarray data. This platform utilizes the computer language R and the limma statistical package to carry out various statistical calculations, such as the empirical Bayes statistics, to identify genes that are differentially expressed between different patient groups.

**Figure 1:**
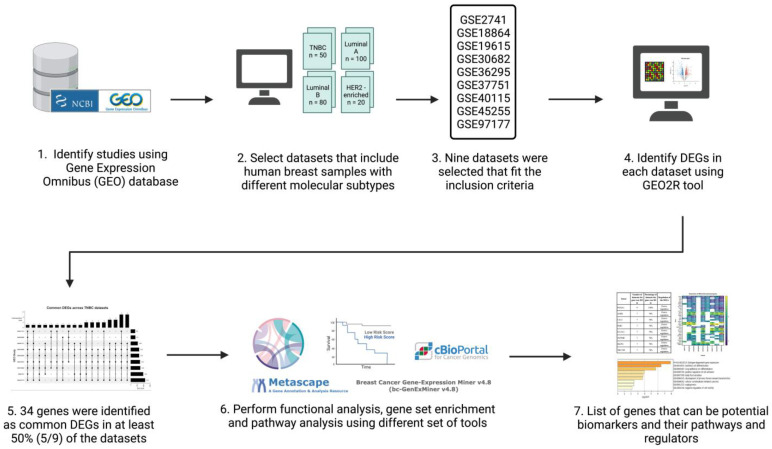
Schematic representation and summary of the re-analysis process of the nine publicly available datasets retrieved from the GEO database. Publicly available data were identified from the NCBI GEO database and analyzed using the GEO2R online tool. The common differentially expressed genes (DEGs) in all datasets were identified and then further analyzed using Metascape, TIMER, cBioPortal, and Breast Cancer Gene-Expression Miner v4.7. Created with Biorender.com accessed on 1 October 2021.

The inclusion criteria for the datasets were: human sample sources, data type was expression profiling by microarray, and datasets had breast cancer patients with TNBC patients included. A total of nine datasets (n = 1027; TNBC n = 207) were used for analysis (Table 1). Patients of each respective dataset were grouped into two groups: a TNBC group and non-TNBC group. Figure 1 illustrates a simplified flowchart of the re-analysis process.

### 2.2. Identification of Differentially Expressed Genes

Each dataset was processed individually to identify DEGs using the GEO2R online tool (https://www.ncbi.nlm.nih.gov/geo/geo2r/, accessed on 1 October 2021). Samples were assigned to groups based on their subtype and analyzed using the standardized parameters of the tool. These standardized parameters include automated log2 transformation of non-transformed data, empirical Bayes method of calculation through the limma statistical package, and adjustment of *p* value using the default Benjamini and Hochberg (false discovery rate) method. *p*-value < 0.05 was used to indicate statistical significance.

To identify consistent DEGs across the nine datasets, all DEGs were intersected using the ‘ComplexUpset’ and ‘UpSetR’ libraries and functions in R studio (R version 4.2.2). Genes that were found to be common in more than 50% of the datasets (5/9 datasets or more) were selected for further analysis.

### 2.3. Gene Ontology and Pathway Analysis

The online database Metascape (http://metascape.org, accessed on 1 October 2021) was used to identify the biological role and functional pathways associated with the common DEGs. Metascape combines a variety of functions including gene annotation, functional enrichment, and membership in over 40 independent databases within a single integrated portal [12]. This tool was used to highlight the significance of the potential connectivity network of our genes and those needed for consideration in order to understand the full biological process [13]. Additionally, this tool streamlines different analysis types instead of searching each database individually. Outcomes include enriched pathways, top transcriptional factors, gene regulators, and protein–protein interactions.

### 2.4. Observing DEG Expression in Patients

To evaluate the expression of the identified DEGs in a clinically relevant cohort, several databases with patient genomic data were used to analyze these DEGs. Patients in these databases can be classified based on their tumor type and subtype. Such databases include the Breast Cancer Gene-Expression Miner v4.7 and the Kaplan-Meier plotter (https://doi.org/10.1016/j.csbj.2021.07.014, accessed on 1 October 2021) to identify the survival of patients based on the expression of selected DEGs.

The cancer genomic database cBioPortal (https://www.cbioportal.org/, accessed on 1 October 2021) was used to identify the survival of patients based on mutations in these common DEGs, as well as to visualize the expression of these genes. cBioPortal hosts multiple cancer databases and/or datasets, and for the basis of this analysis, the TCGA PanCancer atlas was used.

The webserver “TIMER”, an inclusive reserve that analyzes immune infiltrates across various cancer types, was used to evaluate the diagnostic and prognostic value of those specific genes, as well as identify the top immune infiltrates in the breast cancer datasets in relation to these genes.

## 3. Results

**Figure 2:**
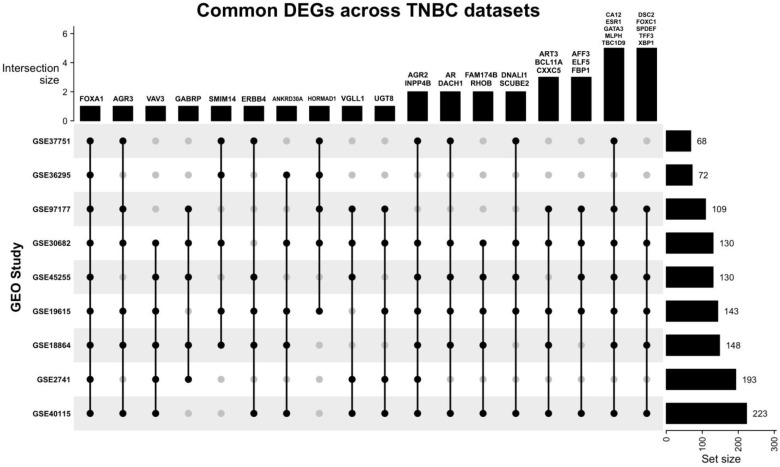
Upset plot illustrating the common DEGs across all nine datasets. Most datasets share at least one DEG with other datasets. FOXA1 is the only DEG that is present in all datasets, and the study GSE30682 has 33 of the 34 common DEGs.

**Figure 3:**
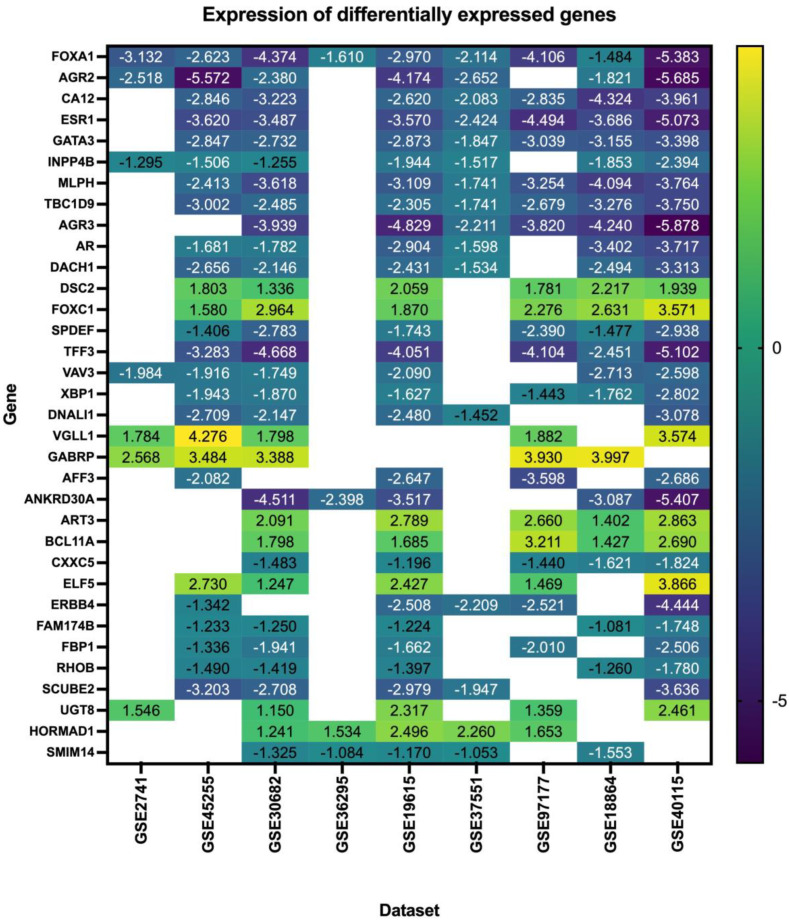
Log fold change of the differentially expressed genes in triple–negative breast cancer across nine datasets.

Our search yielded nine datasets that met our criteria, with a total of 1350 patient samples across the nine datasets. The re-analysis of these datasets revealed a total of 1217 DEGs in all datasets (Supplementary Files S1–S9), 34 of which are consistent across five of the nine datasets (50%) (Figure 2, Table 2, Supplementary File S10), with these genes being either up- or down-regulated. Of the significant and common 34 genes, 26.4% of the genes (n = 9) were up-regulated, and the remaining 73.5% (n = 25) were down-regulated. FOXA1 was the only consistently down-regulated gene across all nine datasets. The log fold change of the 34 DEGs is represented in Figure 3.

### 3.1. Survival Rates of TNBC Patients Are Affected by FOXA1 Expression

**Figure 4:**
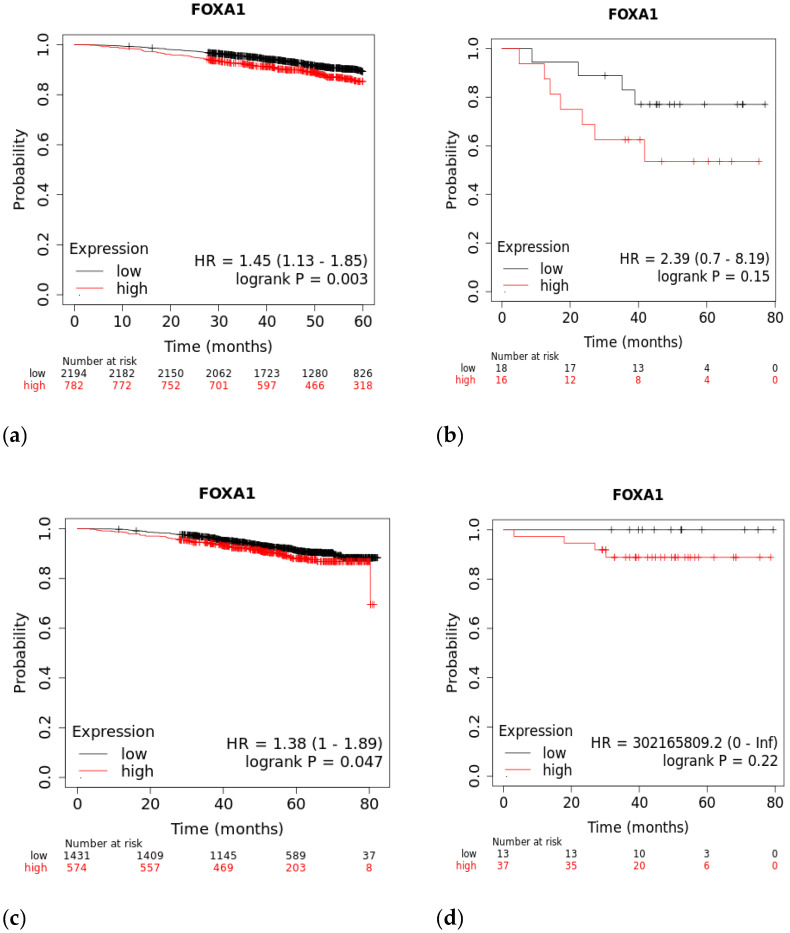
The effect of FOXA1 expression on survival rates. (**a**) Overall survival rate of high vs. low FOXA1 expression in BC patients for the first 60 months since diagnosis; those with high expression rates had lower survival rates. (**b**) Overall survival rate of high vs. low FOXA1 expression in TNBC patients. (**c**) Overall survival rate of high vs. low FOXA1 expression in ER+ and PR+ BC patients. (**d**) Overall survival rate of high vs. low FOXA1 expression in HER2+ BC patients. Created by Breast Cancer Gene-Expression Miner v4.7.

**Figure 5:**
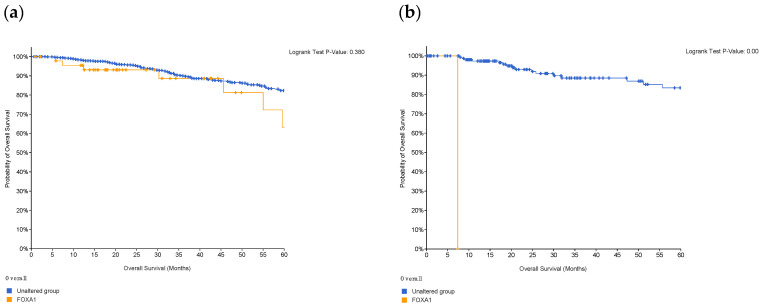
Survival of patients with FOXA1 mutations in (**a**) breast cancer, and in (**b**) patients who are classified as TNBC. Survival decreases in the presence of mutations in the gene; however, in TNBC patients, only one had the mutation and, therefore, this is not of statistical significance. Created by cBioPortal.

A Kaplan–Meier plot was used to test the FOXA1 regulation effect on the survival rate of all breast cancer patients (n = 2976). Patients with high and low FOXA1 expression were compared at a follow-up threshold of five years. Figure 4a reveals that high expression of FOXA1 is associated with a worse prognosis. This low prognosis was consistent when each subtype of breast cancer—TNBC (n = 126), ER/PR–positive (n = 2005), and HER2-positive (n = 30)—was analyzed individually, as shown in Figure 4b–d. Furthermore, another analysis of the TCGA breast cancer dataset from cBioPortal showed that patients with a mutated FOXA1 had a lower survival rate than those without a FOXA1 mutation (Figure 5). However, it is of importance that only one TNBC patient had a mutated FOXA1 in this dataset.

### 3.2. Functional Analysis of the Common DEGs Reveal the Involvement of Estrogen-Dependent Gene Expression Pathway and Related Genes

**Figure 6:**
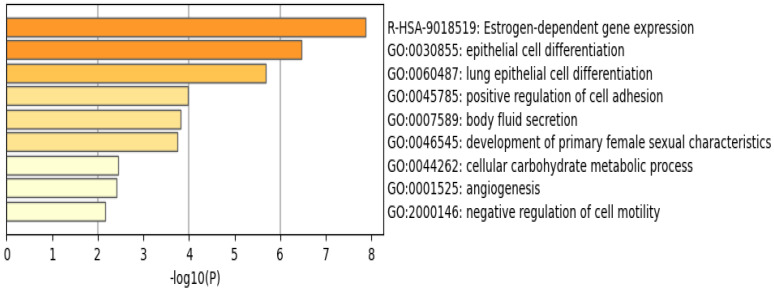
Enriched pathways of the 34 common DEGs using Metascape reveal that estrogen–dependent gene expression is the most significant and enriched pathway. Created by Metascape.

**Figure 7:**
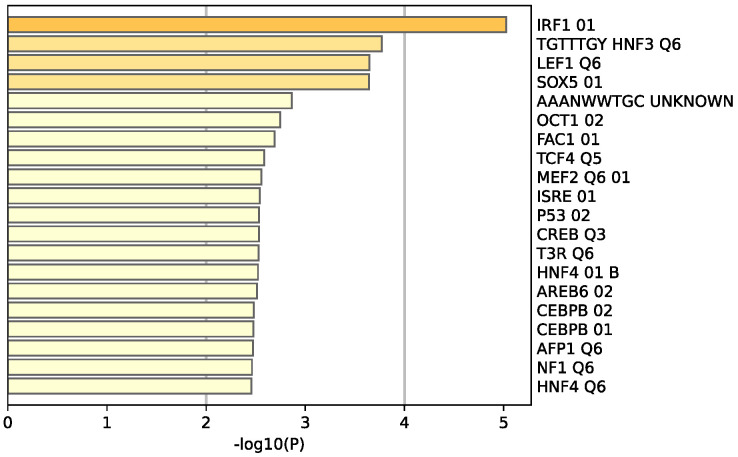
Enrichment analysis of the 34 common DEGS reveal 20 different transcriptomic target factors, with IRF1 being the most common target of these genes. Created by Metascape.

The functional pathway analysis of the 34 common DEGs performed via Metascape provided the pathways these genes were associated with (Figure 6). The most enriched pathway is that of estrogen-dependent gene expression followed by epithelial cell differentiation. Furthermore, the majority of these genes appear to be regulated by the transcription factor interferon regulatory factor 1 (IRF-1), as illustrated in Figure 7.

**Figure 8:**
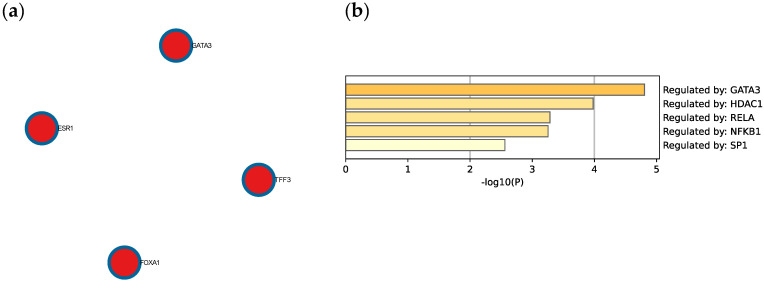
(**a**) Protein–protein interaction network and MCODE components identified in the TNBC significant genes list. (**b**) Summary of enrichment analysis according to gene regulatory functions of the 34 identified DEGs. Created by Metascape.

The Molecular Complex Detection (MCODE) algorithm was utilized to identify densely connected network components. Four genes (GATA3, FOXA1, TFF3, and ESR1) were found to be involved in protein–protein interactions (Figure 8a). Extended enrichment analysis showed that most of the genes were regulated primarily by GATA3, as shown in Figure 8b.

### 3.3. GATA3 Is Down-Regulated in TNBC Which Leads to Poor Survival

**Figure 9:**
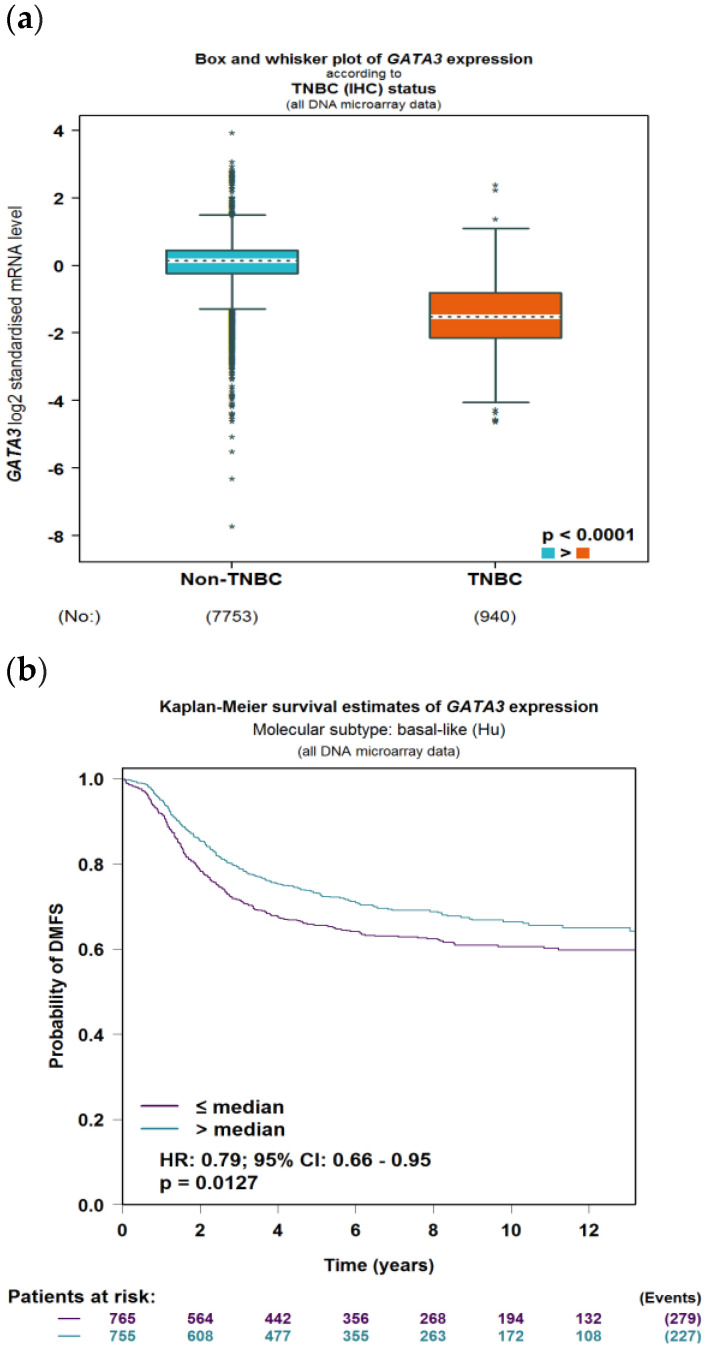

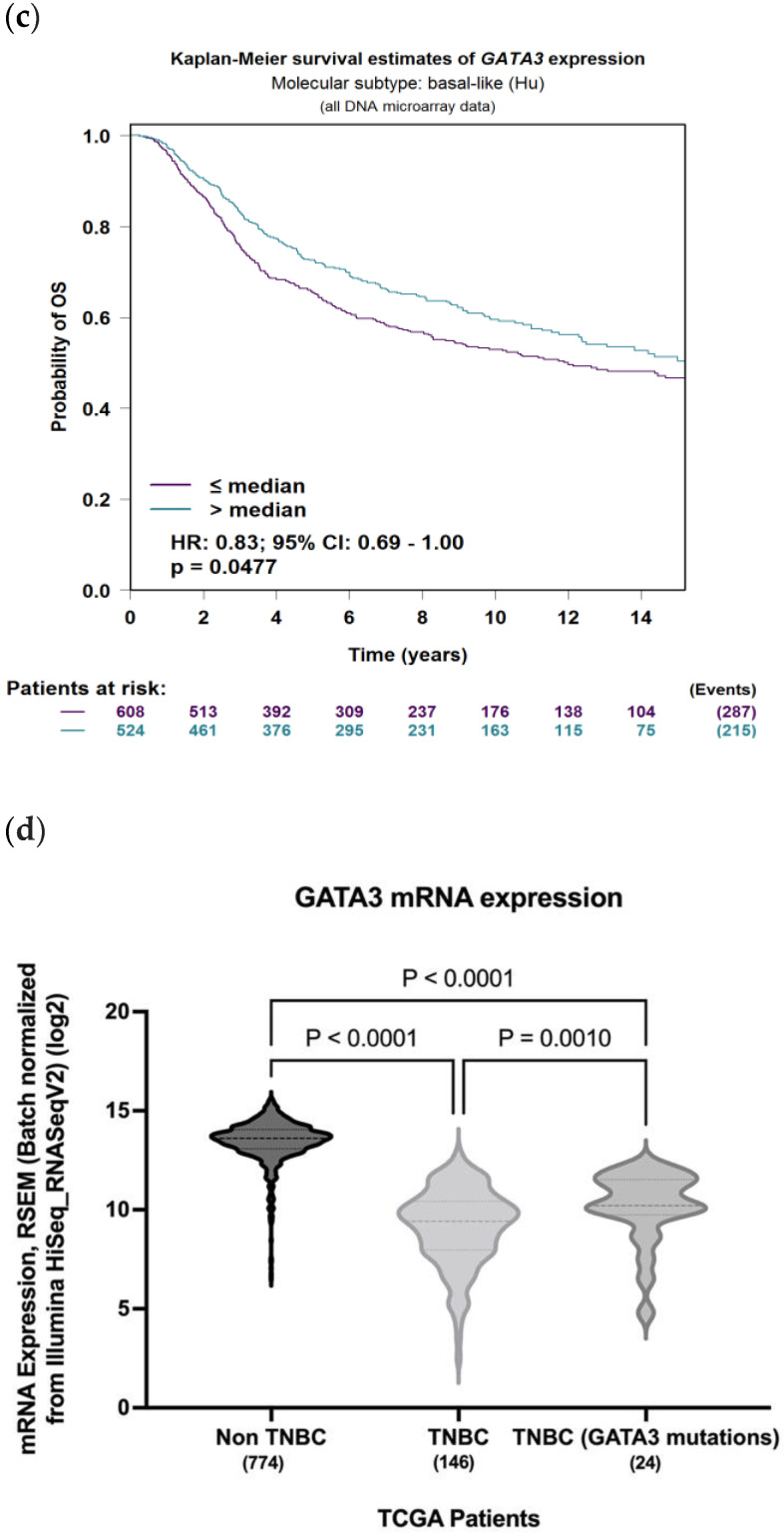
GATA3 expression between breast cancer subtypes. (**a**) GATA3 expression in TNBC vs. non-TNBC. (**b**) Distant metastasis-free survival (DMFS) in TNBC according to GATA3 expression. (**c**) Overall survival in TNBC according to GATA3 expression. (**d**) GATA3 expression in the Cancer Genome Atlas (TCGA) dataset patients obtained from cBioPortal, showing expression in non-TNBC patients, TNBC patients, and TNBC patients with a GATA3 mutation. Created by Breast Cancer Gene-Expression Miner v4.7.

To evaluate the significance of GATA3 expression in terms of diagnostic and prognostic values across the four subtypes of breast cancer in a larger number of patient datasets, Breast Cancer Gene-Expression Miner v4.7 was used. Our analysis revealed a significant decrease in the expression of GATA3 (*p* < 0.0001) in TNBC patients in comparison to non-TNBC patients, as illustrated in Figure 9a. Furthermore, GATA3 expression and nodal involvement in breast cancer were not correlated with each other (*p* = 0.4288), as illustrated in Figure 9b. TNBC with low GATA3 mRNA expression also had a lower distant metastasis-free survival rate as well as a decreased overall survival rate, as shown in Figure 9c,d, respectively. Furthermore, TCGA patient data show that GATA3 is only mutated in 14% of the TNBC patients, with most of these mutations being amplification, while in non-TNBC patients, GATA3 mutations occur in 16% of patients and there are different types of mutations in these patients such as in-frame mutations, splice mutations, and truncating mutations. Consequently, GATA3 expression is higher in non-TNBC patients, followed by TNBC patients with GATA3 amplification and TNBC patients without any GATA3 mutations.

### 3.4. Three Immune Cell Types Are Found in the Tumor Site

Immune cell infiltrates analyzed with the web server “TIMER” reveal that there are three immune cell populations that are particularly involved with GATA3 expression in BRCA-Basal breast cancer are myeloid dendritic cells, neutrophils, and macrophages, as illustrated in Table 3. These immune infiltrates play a role in the innate immune response. The presence of antigen-presenting cells such as macrophages and dendritic cells in the immune microenvironment of the tumor plays a role in tumor progression.

## 4. Discussions

### 4.1. Most DEGs Are Down-Regulated in TNBC Which Can Be Attributed to Poor Prognosis

Within the nine TNBC datasets, there were 34 genes that were consistently differentially expressed, with the majority of these genes being down-regulated, and only nine genes being up-regulated. FOXA1 is the only gene that was down-regulated in all datasets analyzed and has been linked with poor prognosis.

Many of these genes have different roles and functions in breast cancer that affect tumor survival and response to therapy. The R-HSA-9018519 estrogen-dependent gene expression pathway involves the following DEGs: ERBB4, ESR1, GATA3, FOXA1, TFF3, CXXC5, ELF5, and VAV3, with four of these genes being involved in protein–protein interactions and regulated by GATA3. Furthermore, this pathway includes the reaction ‘R-HSA-9018494: FOXA1 and GATA3 bind TFF genes’ that utilizes both FOXA1 and GATA3 to aid oncogenesis and metastasis [14]. The most enriched transcription factor target is IRF1, a transcription regulator and tumor suppressor, which activates genes in both innate and acquired immune responses.

### 4.2. FOXA1 Can Increase Malignancy in Breast Cancer

FOXA1 is a transcriptional factor that plays an important role in hormone signaling in both breast cancer and normal breast tissues [15]. It has been shown that low expression of FOXA1 can increase malignancy and cancer stemness [15]. This gene has been used as one of many subtyping markers in the identification of triple-negative breast cancers [15]. Knock down studies have shown that reduction or deletion of FOXA1 decreases apoptosis and accelerates cell proliferation, which can explain the aggressive nature of TNBC and its worsening prognosis [15]. Furthermore, it has been shown that loss of FOXA1 expression is associated with worse survival and increased expression is an indicator of good prognosis [16,17]. Our re-analysis (Figure 4a) links increased expresion with lower survival rates in the first 60 months for breast cancer patients, which contradicts previous findings. However, when comparing the high and low expression of FOXA1 in TNBC patients, there is no statistical difference. On the other hand, the difference in FOXA1 expression is statistically significant in ER+/PR+ breast cancer. This indicates that FOXA1 might have a different mechanism in TNBCs compared to other sutypes.

### 4.3. GATA3 Is a Major Transcription Factor That Is Found in Many Breast Cancer Subtypes

GATA3 is a transcription factor that is involved in the embryonic development of different types of tissues as well as in inflammatory and humoral immune responses. It is a potent regulator of the tumor microenvironment and plays a role in the proper functioning of the endothelium layer in various types of blood vessels. GATA3 has been proven to be affected in multiple breast cancer subtypes, such as its high expression in the Luminal A subtype due to its strong association with estrogen receptor expression [18]. As seen in our analysis, GATA3 also regulates other DEGs that are affected in TNBC. Due to the strong link between GATA3 and ER expression, high GATA3 levels observed in immunohistochemistry can be used as a positive prognostic method and are linked with favorable pathological features such as positive ER status [19]. This is in line with our results (Figure 9), which link low GATA3 expression with a lower overall survival and distant metastasis-free rate. On the other hand, the lack of an ER receptor in TNBC also reduces GATA3, which is reflected in immunhistochemistry staining sensitivity and is linked with a worse prognosis, distant metastasis-free survival rate, and overall survival [20]. A study carried out in 205 TNBC samples that were divided into five molecular subtypes showed that GATA3 is categorized with a negative stain score (staining intensity x proportion) in 74.6% of all samples [21]. On the other hand, the rate of focal positivity was significantly higher in one of the molecular apocrine subtypes, at 73.9% [21].

However, GATA3 can still stain positive in TNBC, and can be useful when used as a diagnostic and prognostic measuring tool when characterizing metastatic tumors of unknown origin, which is also demonstrated in Figure 9 [22]. This has been supported with a systematic evaluation conducted by Ashley et al., which demonstrated a 44% stain positivity rate of GATA3 across 44 TNBC patients at a staining threshold of 5% [23], and another study revealing a 66% positivity rate when the staining threshold was 1% [24].

Furthermore, mRNA expression of TCGA patients reveals that non-TNBC patients had higher GATA3 expression compared to TNBC patients (Figure 9d). In TNBC patients, those with mutations in their GATA3 had a significantly higher expression (*p* = 0.001) than those without any mutation in their GATA3. These TNBC patients with the GATA3 mutation—all of which are amplification—had a higher survival rate than those without the mutation, which supports the idea that increased GATA3 expression increases survival.

### 4.4. Estrogen-Dependent Gene Expression Plays a Vital Role in Breast Cancer

The R-HSA-9018519 estrogen-dependent gene expression pathway has been shown to be involved in most of the 34 DEGs identified in this study (Figure 6). This is consistent with results from a study published by Treeck O et al., highlighting the effect of estrogen on TNBC, a breast cancer subtype that does not express the estrogen receptor, and yet plays a vital role in pathogenesis [25]. ERα—estrogen receptor alpha—is a major driver of about 70% of breast cancers, with TNBCs being responsive to ERα-independent pathways, which are involved in pathogenesis. A study using a TNBC experimental metastasis model comparing ovariectomy and estrogen supplementation showed that ovariectomy is 56% more efficient in decreasing the frequency of brain metastasis [25]. In addition to ovariectomy, the aromatase inhibitor letrozole reduced the frequency of large lesions by 14.4% in the estrogen control [25]. Another study demonstrated that elevated levels of circulating estrogens were enough to stimulate the development and progression of ERα-negative cancers [26].

The mentioned studies demonstrate that estrogen can act on cells that are distinct from cancer cells, promoting angiogenesis via a systemic pathway by promoting mobilization and recruitment of bone marrow stromal-derived cells in TNBC. This, along with our results (Figure 6), illustrates how these DEGs can serve as biomarkers for TNBC, and how estrogen plays a pivotal role in the pathophysiology of TNBC regardless of the estrogen receptor expression.

### 4.5. GATA3, ESR1, TFF3, FOXA1 Interaction

Of the genes involved in the estrogen-dependent pathway, four genes have been found to be involved in protein–protein interactions. These genes are GATA3, ESR1, TFF3, and FOXA1. ESR1, also known as NR3A1 (nuclear receptor subfamily 3, group A, member 1), is one of the two main types of estrogen receptors. TFF3 is a secretory protein that has multiple and diverse functions such as protection of the mucosa, thickening of the mucosa, and increasing epithelial healing rates [27]. TFF3 has not been well defined, yet closely resmbles the gene TFF1. Some of the properties of TFF1 include inhibition of cell growth, colony formation, and migration and invasion of breast cancer cells in vitro [28].

A study showed that FOXA1 enhances the response to estrogen due to its regulatory properties on the ER binding of the promoter region of its targets [29,30]. The expression of FOXA1 is regulated by GATA3, which, in turn, enhances the expression of the estrogen receptor in epithelial cells [31]. Therefore, if one gene is down-regulated, it will negatively influence genes downstream, which is seen in this study where GATA3 is down-regulated and, therefore, the genes affected by it are also down-regulated. This is indicated in cancer cells that have GATA3 depletion, where there is decreased ESR1-binding affinity, which, in turn, decreases the expression of FOXA1 [32].

While not being involved in the same pathway, TFF1 mRNA expression was correlated with that of FOXA1, GATA3, ESR1, XBP1, and MYB. Additionally, breast cancer patients with a positive ER expressed TFF1 higher than those who were negative for ER [33]. This shows a correlation between TFF1 and the status of estrogen receptor, as seen in the down-regulated TFF3 in TNBC patients. While possessing many genetic interactions, the novel variant TFF3 remains understudied, and further evaluation is required to validate its role and effect in TNBC.

### 4.6. IRF1 Is a Major Transcriptional Factor Target

IRF1 is a transcriptional factor regulator and tumor suppressor that involves immune responses to pathogens such as bacteria and viruses, as well as playing a role in cell proliferation and DNA damage response. This protein represses the transcription of other genes such as by regulating the transcription of INF and INF-induced genes (provided by RefSeq, August 2017). Many of the DEGs identified in this study appear to be associated with this transcription factor.

IRF1 exerts an anti-oncogenic and anti-proliferative effect by its ability to induce the expression of cell growth down-regulatory target genes [34]. Genes targeted by IRF1 include protein kinase R (PKR) and signal transducer and activator of transcription (STAT) and (STAT1) in the Janus kinase (JAK)-STAT pathway. The JAK-STAT pathway signaling is a pathway whose dysregulated activation is known and recorded in many types of tumors and is being studied as a molecular target for cancer therapeutics [35].

The enrichment analysis carried out in this study has shown that the DEGs identified in TNBC are linked to IRF1, a protein that has been considered a potential diagnostic and prognostic biomarker for recurrence-free survival in patients with colorectal cancer by some studies [36]. Therefore, not only can IRF1 be used as a biomarker, but these DEGs can be used as well.

### 4.7. Immune Cell Involvement in Triple-Negative Breast Cancer Can Lead to Better or Worse Prognosis

The tumor microenvironment is an important and variable aspect in the progression of breast cancer. Both the innate and adaptive immune systems with a variety of immune cells are involved in breast cancer [37].

In this study, ‘TIMER’ identified three types of immune cells that were involved in the pathogenesis of TNBC based on the gene expression of GATA3: myeloid dendritic cells, neutrophils, and macrophages. Myeloid dendritic cells were shown to be the most significant immune cell infiltrate. Dendritic cells are a major part of innate immunity and are linked to adaptive immunity through their antigen-presenting properties [38]. A study conducted by Gabrilovich et al. demonstrated the presence of a defect in the dendritic cells of cancer patients, citing that these cells were not effectively presenting antigens [39].

Furthermore, macrophages appear to be significantly involved in basal-like breast cancer based on the TIMER analysis. Macrophages, like dendtritic cells, are antigen-presenting cells and an important part of the innate system. There are two subtypes of macrophages, M1 and M2, which exhibit both inflammatory and anti-inflammatory properties [40]. Breast cancer polarizes macrophages to the M2 form, which is the subtype that promotes tumor growth and cell proliferation [40]. Tumors displaying this subtype of macrophages are often associated with unfavorable prognosis, and favoring features such as nodal involvement and metastasis [41]. Our results demonstrate three immune cell populations involved in breast cancer in relation to GATA3 expression, and the importance of this gene towards the immune microenvironment. Similarly, a study conducted by Dieci M et al. demonstrates the importance of immune infiltrations’ involvement in breast cancer and the possibility of their use as potential biomarkers [38].

### 4.8. Clinical Implications

Significant key information about tumors can be obtained from the identified biomarkers, especially as a prognostic tool. Patient prognosis can be evaluated according to the biomarkers present in tumor DNA, which is an advisable screening option due to the lack of well-defined molecular targets that make cytotoxic chemotherapy the only treatment option for TNBC patients [42]. In order to avoid such harsh treatment plans, understanding genetic biomarkers could provide a platform for new diagnostic and therapeutic options specifically designed to target TNBC, with the expression of selected markers being used as identifiers for the ideal course of treatment and response to such treatments. For example, GATA3 can be used to evaluate response to hormonal treatments targeting the estrogen receptor pathways.

Another implication is that four of the genes identified in this study—AGR2, AGR3, TFF3, and SCUBE2—have protein products that are secreted in the blood by breast cancer. This can lead to the use of non-invasive methods such as blood tests for preliminary diagnosis before tissue biopsies, which can lead to more tests being conducted and earlier detection. Another benefit of using blood tests can include increased testing for more robust monitoring of the disease, such as taking a test before and after treatments.

### 4.9. Strengths and Limitations

This study was carried out using in silico methods and tools, which revolve around the use of publicly available transcriptomic data to mimic in vitro studies. A benefit to in silico analysis is the data accessibility and cost-effectiveness of this method, with a large body of patient data and databases available. This approach could reduce the time for the conceptualization of a hypothesis before going into in vitro testing, as well as identifying targets for in vitro testing and validation. Furthermore, this in silico study helped create a shortlist of potential genes that are involved in TNBC, which can be studied further and used to generate different hypotheses.

However, these in silico studies need in vitro validation to confirm any final conclusions. Furthermore, in silico data can result in contradicting results due to the varying patient types and accompanying clinical information in each dataset. This has been observed in some of our data, as cBioPortal has only one TNBC patient with a FOXA1 mutation, compared to Breast Cancer Gene-Expression Miner v4.7, which has several TNBC patients with high or low expression. Therefore, the use of one database is not enough and several tools need to be used, as we have in our re-analysis.

## 5. Conclusions

In conclusion, our study identified 34 DEGs in TNBC compared to the other subtypes of breast cancer. The generated shortlisted genes could be used in clinical settings as biomarkers to detect TNBC at an early stage and improve the overall prognosis of the patient as well as aiding in their treatment course. This in silico analysis study demonstrated the various physiological effects of the genes involved in TNBC such as estrogen-dependant pathways, which provide possible alternative targeted treatment options as compared to the standard non-specicific options currently available. Given that this study is an in silico analysis, we had access to a limited number of patients in certain subtypes of disease, and need further validation. We believe that our findings could provide advancements in the field of TNBC, and we encourage future in vitro and in vivo studies to further solidify the validity of these results.

## Figures and Tables

**Table 1:** List of breast cancer studies used in this analysis from NCBI GEO database. TNBC: triple-negative breast cancer.

GEO Accession Number	Study Title	Samples	PMID
GSE2741	Breast Tumor’s study	TNBC = 3 Non-TNBC = 8 Total samples = 11	16230372
GSE45255	Expression Profiles of Breast Tumors from Singapore and Europe	TNBC = 15 Non-TNBC = 124 Total samples = 139	23618380
GSE30682	Search for a gene-expression signature of breast cancer local recurrence in young women	TNBC = 58 Non-TNBC = 285 Total samples = 343	22271875
GSE36295	Transcriptomic analysis of breast cancer	TNBC = 11 Non-TNBC = 27 Total samples = 38	27177292
GSE19615	Integrated genomic and function characterization of the 8q22 gain	TNBC = 28 Non-TNBC = 87 Total samples = 115	20098429
GSE37751	Molecular Profiles of Human Breast Cancer and Their Association with Tumor Subtypes and Disease Prognosis (Affymetrix)	TNBC = 14 Non-TNBC = 47 Total samples = 61	30501643
GSE97177	Genome-wide multi-omics profiling reveals extensive genetic complexity in 8p11-p12 amplified breast carcinomas [expression]	TNBC = 9 Non-TNBC = 44 Total samples = 53	29844878
GSE18864	Tumor expression data from neoadjuvant trial of cisplatin monotherapy in triple-negative breast cancer patients	TNBC = 38 Non-TNBC = 46 Total samples = 84	20100965
GSE40115	Classifications within Molecular Subtypes Enables Identification of BRCA1/BRCA2 Mutation Carriers by RNA Tumor Profiling	TNBC = 31 Non-TNBC = 152 Total samples = 182	23704984

**Table 2:** Differentially expressed genes present in 50% or more of the datasets analyzed. DEG: differentially expressed gene, DE: differentially expressed.

Genes	Gene Name	Number of Datasets the Gene Was DE in	Percentage of Datasets the Gene Was DE in	Regulation of the DEG in TNBC
FOXA1	Forkhead box A1	9	100%	Down-regulated
AGR2	Anterior gradient 2	7	78%	Down-regulated
CA12	Carbonic anhydrase 12	7	78%	Down-regulated
ESR1	Estrogen receptor 1	7	78%	Down-regulated
GATA3	GATA binding protein 3	7	78%	Down-regulated
INPP4B	Inositol polyphosphate-4-phosphatase type II B	7	78%	Down-regulated
MLPH	Melanophilin	7	78%	Down-regulated
TBC1D9	TBC1 domain family member 9	7	78%	Down-regulated
AGR3	Anterior gradient 3	6	67%	Down-regulated
AR	Androgen receptor	6	67%	Down-regulated
DACH1	Dachshund family transcription factor 1	6	67%	Down-regulated
DSC2	Desmocollin 2	6	67%	Up-regulated
FOXC1	Forkhead box C1	6	67%	Up-regulated
SPDEF	SAM pointed domain containing ETS transcription factor	6	67%	Down-regulated
TFF3	Trefoil factor 3	6	67%	Down-regulated
VAV3	Vav guanine nucleotide exchange factor 3	6	67%	Down-regulated
XBP1	X-box binding protein 1	6	67%	Down-regulated
DNALI1	Dynein axonemal light intermediate chain 1	6	67%	Down-regulated
VGLL1	Vestigial-like family member 1	5	56%	Up-regulated
GABRP	Gamma-aminobutyric acid type A receptor subunit pi	5	56%	Up-regulated
AFF3	ALF transcription elongation factor 3	5	56%	Down-regulated
ANKRD3OA	Ankyrin repeat domain 30A	5	56%	Down-regulated
ART3	ADP-ribosyltransferase 3 (inactive)	5	56%	Up-regulated
BCL11A	BCL11 transcription factor A	5	56%	Up-regulated
CXXC5	CXXC finger protein 5	5	56%	Down-regulated
ELF5	E74-like ETS transcription factor 5	5	56%	Up-regulated
ERBB4	Erb-b2 receptor tyrosine kinase 4	5	56%	Down-regulated
FAM174B	Family with sequence similarity 174 member B	5	56%	Down-regulated
FBP1	Fructose-bisphosphatase 1	5	56%	Down-regulated
RHOB	Ras homolog family member B	5	56%	Down-regulated
SCUBE2	Signal peptide, CUB domain and EGF-like domain containing 2	5	56%	Down-regulated
UGT8	UDP glycosyltransferase 8	5	56%	Up-regulated
HORMAD1	HORMA domain containing 1	5	56%	Up-regulated
SMIM14	Small integral membrane protein 14	5	56%	Down-regulated

**Table 3:** Immune cell involvement in basal-like breast cancer.

Cancer	Infiltrates	*p*-Value	Adjusted *p*-Value
BRCA-Basal (n = 191)	Myeloid dendritic cell activated	0.00151881	0.005368
BRCA-Basal (n = 191)	Neutrophil	0.019378582	0.049857
BRCA-Basal (n = 191)	Macrophage	0.006514787	0.019388

## Data Availability

The datasets supporting the conclusion of this article are available in the NCBI repository, (https://www.ncbi.nlm.nih.gov/geo/, accessed on 1 October 2021), with their accession codes in Table 1.

## Supplementary Materials

The following supporting information can be downloaded at: https://www.mdpi.com/article/10.3390/life13020422/s1. Supplementary File S1: GSE2741; Supplementary File S2: GSE18864; Supplementary File S3: GSE19615; Supplementary File S4: GSE30682; Supplementary File S5: GSE36295; Supplementary File S6: GSE37751; Supplementary File S7: GSE40115; Supplementary File S8: GSE45255; Supplementary File S9: GSE97177; Supplementary File S10: Common DEGs.

## Abbreviations

MBRU	Mohammed Bin Rashid University of Medicine and Health Sciences
ER	estrogen receptor
PR	progesterone receptor
HER2	human epidermal growth factor receptor 2
NCBI	National Center for Biotechnology Information
GEO	Gene Expression Omnibus
IRF-1	interferon regulatory factor 1
MCODE	Molecular Complex Detection
DMFS	distant metastasis-free survival
OS	overall survival
PKR	protein kinase R
STAT	signal transducer and activator of transcription
JAK	Janus kinase
NR3A1	nuclear subfamily 3, group A, member 1
HNF-3A	hepatocyte nuclear factor 3-alpha
